# Homes of Stroke Survivors Are a Challenging Environment for Rehabilitation Technologies

**DOI:** 10.2196/12029

**Published:** 2021-06-17

**Authors:** Stefan Rennick-Egglestone, Sue Mawson

**Affiliations:** 1 School of Health Sciences Institute of Mental Health University of Nottingham Nottingham United Kingdom; 2 School of Health and Related Research University of Sheffield Sheffield United Kingdom

**Keywords:** domestic rehabilitation technology, brain injury, stroke, research through design

## Abstract

The design of digital technologies that support poststroke rehabilitation at home has been a topic of research for some time. If technology is to have a large-scale impact on rehabilitation practice, then we need to understand how to create technologies that are appropriate for the domestic environment and for the needs and motivations of those living there. This paper reflects on the research conducted in the Motivating Mobility project (UK Engineering and Physical Science Research Council: EP/F00382X/1). We conducted sensitizing studies to develop a foundational understanding of the homes of stroke survivors, participatory design sessions situated in the home, and experimental deployments of prototype rehabilitation technologies. We identified four challenges specific to the homes of stroke survivors and relevant to the deployment of rehabilitation technologies: identifying a location for rehabilitation technology, negotiating social relationships present in the home, avoiding additional stress in households at risk of existential stress, and providing for patient safety. We conclude that skilled workers may be needed to enable successful technology deployment, systematizing the mapping of the home may be beneficial, and education is a viable focus for rehabilitation technologies.

## Introduction

The design of interactive technologies to support rehabilitation from disability acquired through stroke has been a topic of research since 1991, when Dijkers et al [1] presented a novel robotic system that could guide a stroke patient through a series of reaching exercises, under the hypothesis that repeated use could support the rehabilitation of movement in the affected limb. Research has subsequently expanded to cover a broad range of robotic installations intended to support the rehabilitation of motor abilities [2] and to approaches such as the use of virtual reality content to motivate physical engagement with robotic installations and to ground motor skills relearning in practical examples [3-5]. Many of these installations have been targeted at high-throughput clinical environments where individuals might spend a relatively short period.

Most rehabilitation takes place post discharge, frequently in the home, and this phase has historically been poorly supported by health services [6]. As a result, in recent years, there has been a shift in service delivery from hospital-based rehabilitation to the community. Although rehabilitation would ideally continue until maximum recovery has been achieved [7], the increasing demand for services and financial constraints typically means that service needs often cannot be met, potentially creating a situation in which outcomes for stroke survivors are suboptimal. A radical alternative paradigm, which has been explored through initiatives such as the Expert Patient Programme [8,9], is self-management, which has also been translated into the concept of self-care [10]. The aim of self-care is to help individuals take control of their own health and well-being, potentially supported by services designed to enable this [11].

Evidence suggests that long-term, intense, task-specific, context-specific, goal-oriented, variable, and environmentally enriched poststroke rehabilitation improves function, independence, and quality of life [12,13]. Significant advances in the performance and affordability of information and communication technologies have led to the exploration of its use to support rehabilitation that is clinician led or from a self-management or self-care perspective [14]. Examples include the use of commodity gaming technologies such as the Nintendo Wii or Logitech EyeToy to motivate significant amounts of movement [15,16], the provision of telecare technologies that allow health care practitioners to monitor and guide progress in engaging with rehabilitation activities [17], and the integration of relatively inexpensive force feedback interaction devices to help support someone in engaging in a difficult motor activity, under the hypothesis that repeated assisted completions of activity will lead to long-term improvement in the ability to conduct nonassisted completions [18].

Even at a technological level, the widespread provision of home-based technologies is a very different type of challenge to that of provision for the clinical environment. Home-based deployment of technology might naturally lend themselves to (and necessitate) technologies that are commodified, with a much lower unit price, to facilitate large-scale uptake. As a target for technology design and deployment, the home should not be thought of as a smaller-scale, lower-intensity version of a clinic. Foundational research within the fields of computer-supported cooperative work and human-computer interaction has sought to draw technology designers’ attention to the home as a playful place [19,20]; as an often private place of sanctity and relaxation [21,22]; and as a space with an often complex and nonhierarchical social structure, far removed from the more rigid social structures that might be found in the workplace [23]; hence, in a rehabilitation technology context, it is very different in nature from the more rigid and streamlined nature of the clinical environment. There is an explicit recognition within these fields that the home is inherently a challenging environment for the deployment of digital technologies (eg, see the discussion in the study by Tolmie et al [24]).

Given that brain injuries can lead to profound and varied disabilities and hence inherently raise the difficulty of living in a space [25], the homes of stroke survivors are likely to raise additional challenges specific to the disruption that stroke can cause and which rehabilitation technology designers or deployers should attend to in their work. There may be a danger that rehabilitation technology, if not carefully designed to have a place in the homes of stroke survivors, will simply become another burden, likely to be engaged with infrequently and hence ineffective at supporting rehabilitation. Some practical examples of such barriers have been provided in studies by Axelrod et al [26] and Threapleton et al [27]. To become widespread, integration of interactive rehabilitation technologies into home-based therapeutic practice is likely to require a rich understanding of the challenges that this environment presents for technology development work.

As a contribution to this developing area of inquiry, this paper draws on the work conducted through *Motivating Mobility: Interactive Systems to promote Physical Activity and Leisure for people with limited mobility*, a 3-year research study funded by the UK Engineering and Physical Sciences Research Council. Motivating Mobility was a collaboration among researchers with expertise in psychology, physiotherapy, technology design, and technology deployment.

In Motivating Mobility, we engaged in three phases of work selected to provide a rich understanding of the home and how rehabilitation technology targeted at upper-limb disabilities might find a place in it. This paper integrates results from published and unpublished Motivating Mobility studies to identify challenges in the deployment of rehabilitation technologies into the home environment and implications of these challenges for health care practices.

## Research Approach

Work in the Motivating Mobility study was situated within an emerging approach known as Research through Design, characterized effectively by Zimmerman et al [28], which positions the technology design process as a vehicle to generate knowledge about a setting. Research through Design typically supports the generation of knowledge by loosening financial or temporal constraints that might constrain a more commercially oriented technology design process, and knowledge is generated through reflection on the design process, the information that it draws on, and the decisions that are made within it. Research through Design has been applied in health research, including the work by Thieme et al [29], who reported on lessons learned through the design and integration of bespoke-designed digital artifacts into a secure mental health service.

As a piece of Research through Design, work in Motivating Mobility was structured into three phases. Phase 1 consisted of sensitizing studies that provided initial insights into the nature of the home environment to ground the work of the project. Phase 2 consisted of situated design case studies in which the research team visited the homes of 4 stroke survivors and worked collaboratively to identify technologies that might support rehabilitation and find an effective place in their homes. In phase 3, prototype implementations of technology were deployed into 4 homes, usage was captured through electronic logs, and interviews were conducted to understand how technologies were or were not appropriated. Detailed methods and findings are presented in studies by Egglestone et al [25], Axelrod et al [26], and Balaam et al [30,31]. Ethical approval was obtained in advance from the University of Sussex Research Ethics Committee, and informed consent was obtained in writing before participation in any of the studies that have informed this paper. Participants in design case studies had the right to opt out of the use of photographic material in research publications.

In this paper, we have reexamined the material collected by Motivating Mobility studies to present emergent findings that represent the totality of what we have learned through this work. Our focus was on what we have learned about the challenges of the domestic environment for rehabilitation technology design and deployment.

## Challenges for Domestic Rehabilitation Technologies

### Challenge 1: Identifying a Location for a Rehabilitation Technology

**Figure 1:**
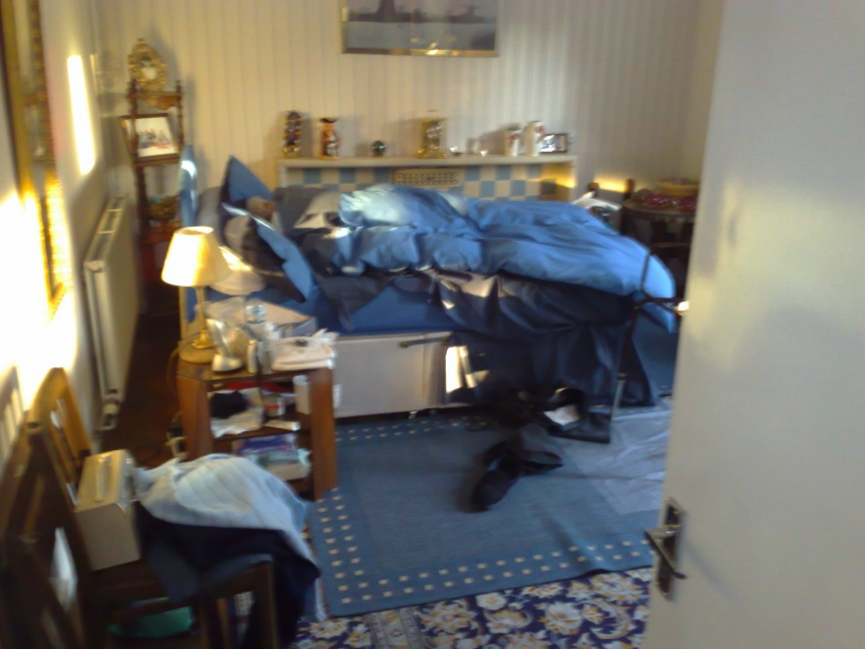
Selected image from photographic sensitizing study. First published in the study by Axelrod et al [26].

Early in Motivating Mobility, a photographic study of the homes of stroke survivors was conducted [26]. Although this can only present a snapshot of the lives of the recruited participants (Figure 1), it does provide graphical evidence for phenomena that are likely to be widely recognizable, such as rooms repurposed because of disability (eg, from a lounge to a bedroom) and surfaces cluttered with possessions because of the difficulty of conducting organizing tasks owing to the acquired disability. Through focus groups conducted with stroke survivors, we learned of participants who had needed to downsize to a smaller property, sometimes through loss of income. Such changes can be deeply distressing.

One implication is that finding a physical space to place digital technologies may be more difficult in the homes of stroke survivors. This does not preclude the integration of technology; we found that participants were often willing to make changes to their homes to incorporate technologies that they believed to have benefits. This suggests that finding an appropriate location is a necessary part of the technology deployment process. It may also implicate work to persuade residents of the benefits of the technology versus the effort of integrating it into their space.

The complexity of finding an appropriate location for a rehabilitation technology may be enhanced by long-term residency in a home, frequently measured in decades, and by the nature of the home as an ongoing project to create a pleasing and stimulating environment [32]. In one of our design case studies, a participant described their home as a 30-year project with every element selected through hours of thought and maintained with love and care. Adding an obtrusive digital technology to such a controlled environment requires careful negotiation and ultimately risking the technology to be unacceptable to the user and hence potentially rejected for deployment. A study by Threapleton et al [27] has raised the possibility of bulky prototype equipment harming recruitment rates to research studies because of its impact on the domestic environment.

**Figure 2:**
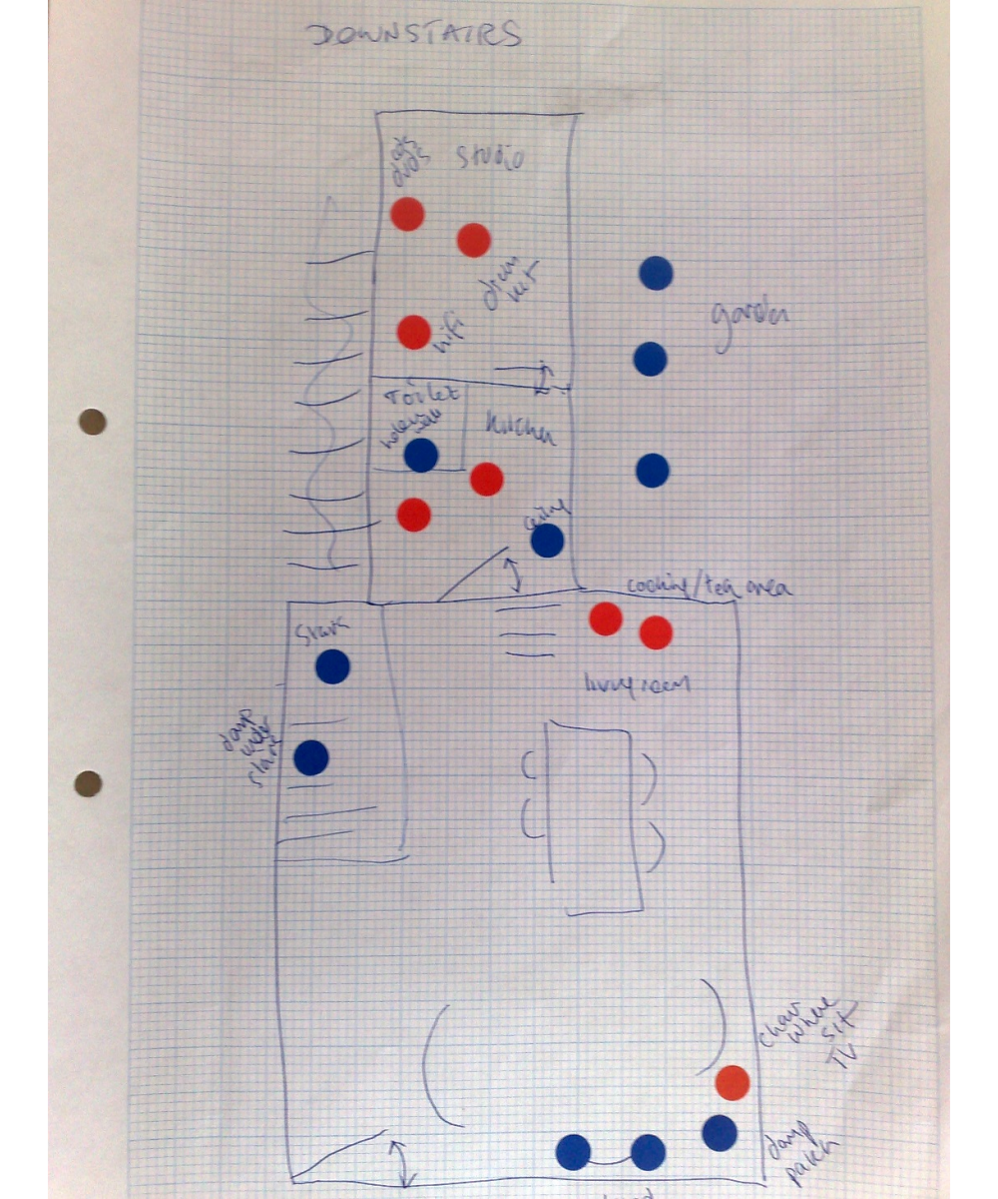
Emotion map of the home of a stroke survivor. First published in the study by Axelrod et al [26].

Further complications might arise from the changed and often heightened emotional reaction to the home, which can be caused by stroke. One example can be seen in an *emotional map* (Figure 2), captured in a sensitizing study, along with an interview explaining its meaning to the contributor [26]. In Figure 2, the red dots indicate physical spaces in the home that provoke anxiety. Red dots in the kitchen were placed there by the participant to indicate residual anxiety caused by this being the location in which the stroke occurred, even though the interview was several years poststroke.

Other examples of heightened emotional reactions to the home include the following:

- A participant who had slept in a spare room for many years poststroke as she had experienced a stroke in her bedroom and had since been unable to reenter this because of the acquired emotional load associated with it.
- Participants distressed by locations associated with practical activities that had become difficult because of the acquired disability (eg, kitchens or utility rooms).

Technology deployment work may need to take into account these heightened reactions.

A substantial body of literature describes integrating elements of the domestic environment directly into rehabilitation technologies. A study by Pridmore et al [33] has described a mixed-reality kitchen environment in which users are encouraged to perform repeated exercises involving the manipulation of tracked physical kitchen artifacts such as kettles as part of relearning practical skills. Although we do not argue that such designs are inappropriate, we would suggest that the often profound emotional changes caused by stroke might make certain locations challenging for technology deployments. Such locations may provide a rich resource for meaningful interactions if handled sensitively and may provide a route toward long-term improvements in quality of life. A technology that supports effective reengagement with a location that induces anxiety might provide substantial benefits to its user. However, if handled naively, for example, if a rehabilitation technology is placed in a difficult location without careful consideration, then they risk underusage or no usage at all of the technology or of creating negative associations of the technology.

Given the challenges described earlier, finding an appropriate place in the home for technology quickly became a central question for our research. In our design case studies, we addressed this through an initial design session where we discussed issues of space and place with our participants. This often led to a specific first proposal for where a technology might be placed, which was then further discussed in subsequent design sessions with a participant.

One notable phenomenon was that our participants sometimes already inhabited a *safe* or *stimulating* space for a substantial proportion of their day, which had been specifically designed to support their well-being. The tactics that we observed in creating these spaces included the use of a comfortable armchair with accompanying photographs, entertainment systems, and necessary physical support (such as armrests), placed in a space in the home that was less likely to induce anxiety. The motivation for creating such spaces seemed to account for and ameliorate the negative effects of the difficulties in mobility acquired through stroke.

**Figure 3:**
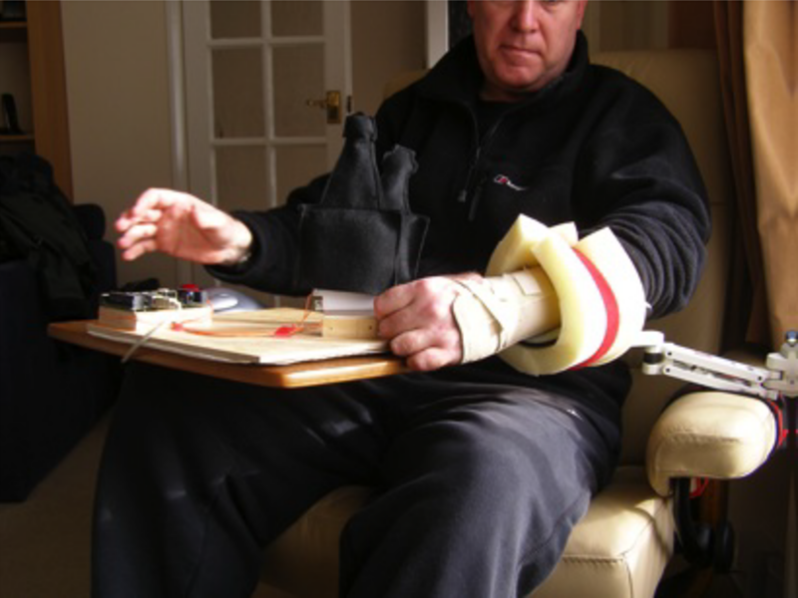
Technology added to a “stimulating space”.

In two of our design case studies, we worked with participants to explore a tactic of appropriating these spaces for the purpose of introducing technology (Figure 3). This involved integrating digital technologies with ergonomic elements to enable the technology to be positioned in the space. Discussions with participants suggested that a possible negative outcome of this approach might be a perceived reduction in the support that the space provided for well-being; this is then a danger which should be seen in light of the discussion around situated anxiety provided earlier.

### Challenge 2: Negotiating Social Relationships

Through sensitizing studies, we learned of the active role that others sharing a home, such as partners, can take in the rehabilitation process. Participants told us of partners who had learned a great deal about stroke and rehabilitation theory to provide more expert support for ongoing rehabilitation efforts. They also told us of partners who had made substantial modifications to the home to support well-being. To learn about the potential role of coresidents in relation to technology, we invited partners to all participatory design sessions and scheduled these sessions to allow for the provision of input from partners wherever possible. For one design case study, with a stroke survivor in her 30s, parents were also invited, as they played a significant and active role in her care. All prototype deployments were in shared environments, and we explicitly included coresidents in the follow-up interview process.

In addition to opportunities, we observed that the presence of others in the same household could create contention over the usage of space. In the design case study illustrated in Figure 3, we used a television as an output device for a piece of situated technology because of its proximity to a stimulating space; however, this introduced a contention around the usage of the television, which was seen to substantially reduce engagement with our technology.

**Figure 4:**
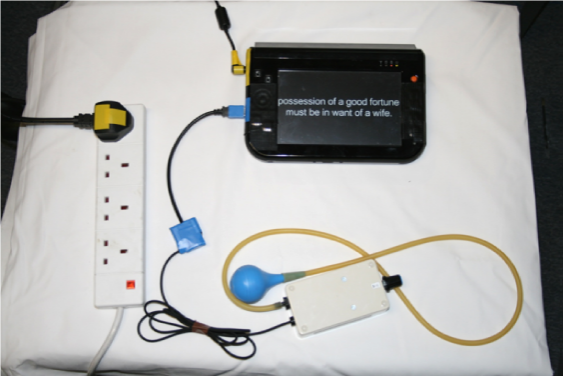
The Rehab Reader. First published in the study by Balaam et al [30]

One notable example of the way in which a partner might shape an interaction with a technology was provided in a design case study that we called the *Rehab Reader* [30]. The study used a tablet PC attached to a commodity squeeze sensor. Various electronic books were loaded onto the PC, and the sensor was configured so that a squeeze allowed for progressing through the book, one paragraph at a time. In designing this technology, our intention was for repetitive exercise to be conducted as a side effect of a meaningful and enjoyable activity, and hence for significant quantities of exercise to be conducted—quantity of exercise is currently believed to be a key determinant of the effectiveness of motor rehabilitation. Our participant Irene (not her real name) had lost the ability to read books because of the eyesight damage caused by stroke, and the flexibility of a digital device allowed us to present text in a size with which she could engage. As such, it gave renewed access to a hobby that she had previously valued. A photograph of the prototype implementation of the Rehab Reader is provided in Figure 4.

A prototype was deployed for a period of 7 months, and a total of 6621 grasp and release exercises were conducted during this period. Substantial grasp and release recovery was noted in the hand used to control the device. However, a contention emerged between Irene and her partner about the meaning of the device, and hence how it should be used. Irene saw the prototype as a leisure device, with exercise occurring as a side effect. Her partner saw it as an exercise device, similar in nature to how he saw a Nintendo Wii owned by a couple. Irene’s partner pressured her to use it at fixed and regular times during the day, so as to perform a reliable amount of exercise. She wanted to use it when she felt like it, and this contention in meaning caused arguments within the couple. It is possible that this contributed to the overuse of the device, as described in challenge 4. Although this is a very specific scenario, we present it to draw attention to the importance of understanding the social nature of interaction with rehabilitation technologies. Others in a social setting are key actors who need to be understood as part of a rehabilitation technology deployment and who can play a role in relation to it, which ranges from supportive to disruptive. As such, their engagement may need to be managed.

**Figure 5:**
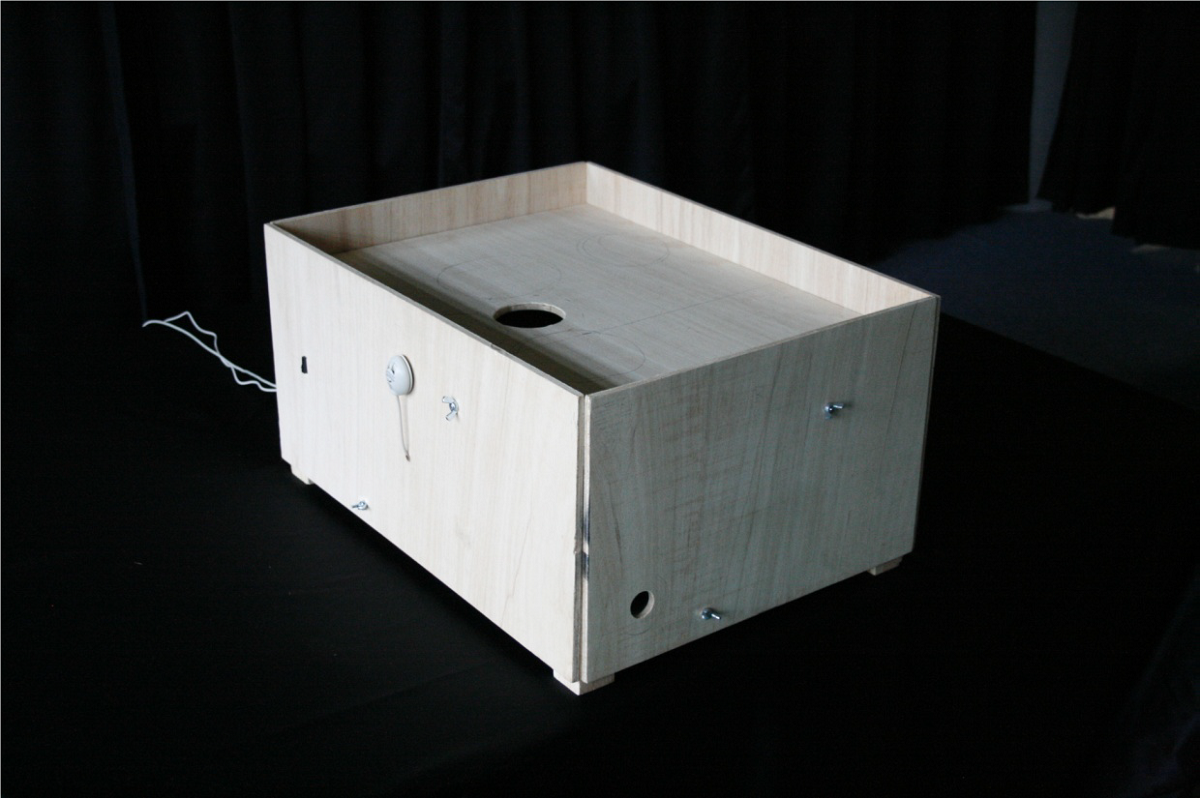
The Ball FUNnel—prototype of a child’s toy.

One of our least successful prototype deployments involved a novel children’s toy specifically designed to allow a mother who had experienced a stroke in her 30s to perform rehabilitation motor exercise while playing with her young child, a key person on the social side of her existence (Figure 5). The tactic here, discussed in great detail with our participant before construction of the prototype, was essentially to use play with the child as a motivator for engagement. However, the outcome of this choice was that engagement was tied to the sustained interest of the child, and when the child got rapidly bored with the toy, then this motivation, and hence any kind of physical exercise, was ended. This is a case study that suggests some degree of caution when attempting to appropriate the social context into technology design because of the introduction of a dependency on an (essentially unpredictable) individual.

### Challenge 3: Avoiding Additional Stress in Households at Risk of Existential Stress

We learned through our sensitizing studies of the almost intolerable levels of existential stress that could be experienced in the homes of stroke survivors [25]. Stress might be caused by the need to make rapid changes to the organization of the home, with partners picking up domestic duties that they might be unprepared for. Difficulties are often accentuated by their own health conditions. Stroke frequently had a substantial negative impact on the social lives of both members of a couple; hence, stress was increased through the loss of social interaction. It could also be accentuated by the need to move to a smaller residence for financial reasons and the sometimes poor quality of social care provided by the local government. Existential stress was universal across participants and at the margins of what could be coped with.

A household with continuing high levels of stress around their everyday existence may be a difficult environment to deploy new technologies, especially if those technologies require additional effort to learn about, engage with, or maintain in a working state. Potentially, rehabilitation technologies that do not take into account or which increase the already high levels of stress may be more likely to fail. In Motivating Mobility, we adopted a tactic of not building technologies that depended upon network connections, in the belief that having to maintain the functionality of a network connection would likely lead to excessive stress and hence lack of use of the technology. We note that our work was done in a period when domestic technology was frequently discussed as being unstable and requiring skilled work to maintain (refer to Tolmie et al [24] for a contemporaneous ethnographic study of the work to keep networking working).

A second technological contributor to domestic stress might include the clutter of cabling, chargers, displays, and interaction devices that make up a technology deployment. Technological clutter might be thought of as particularly problematic if domestic residents are already struggling to keep on top of basic domestic tasks such as cleaning. Much of our participatory design work focused on identifying technologies that were as self-contained as possible, with as little clutter as could be achieved. This involved working with participants to find appropriate spaces in their homes for necessary components such as chargers and creating devices that were engineered to require as little expert maintenance as possible. Producing technologies that did not add to clutter sometimes involved substantial design and engineering work. However, none of our prototype technologies were rejected due to the clutter that they created, so we feel that this effort added substantial value.

### Challenge 4: Providing for Patient Safety

To support the design of interventions that were effective, design case studies were conducted as an active collaboration between interaction designers familiar with the domestic environment, software engineers, and physical therapists familiar with stroke treatment. An open question was the implications for future health care practice of creating effective domestic rehabilitation technologies; in this context, the research therapists working on the project modeled how a practicing therapist might respond in the future and allowed us to learn, on a small scale, what a technologically augmented therapist role may look like.

In practice, therapeutic intervention in design case studies has two principal roles: identifying exercises that might provide rehabilitation benefits if conducted regularly and monitoring deployments for effectiveness and safety. The latter involved regular contact between a technology user and a therapist, conducted either in person or by telephone, as appropriate. The therapist talked about the usage of technologies and any manifestations of physical problems. In one case, this process led to a modification to a prototype implementation of a piece of technology that had already been deployed, in order to correct an ergonomic problem caused by it being used in a different location than the one planned, meaning that it was causing discomfort to the user, with usage potentially detrimental to rehabilitation.

The most heavily used of our prototypes was the Rehab Reader, described in detail earlier. Regaining the ability to read books provided such a benefit to the participants that they used it for many hours per week. Early in the intervention, they reported to their appointed therapist experiencing some pain in the eye affected by stroke and were advised to reduce their usage while this had a chance to adjust. The therapist also visited to provide alternate physical interaction devices to support the avoidance of repetitive strain injuries, observed the usage, and offered guidance on appropriate and inappropriate physical positioning of the device in order to avoid encouraging movements that were not of a high-quality nature.

It is conceivable that some of the work of a physical therapist might be encoded in rehabilitation technologies, especially given the recent advances in the capabilities of Artificial Intelligence systems. However, what the above examples highlight is that any deployment of a rehabilitation technology is likely to need some engagement from an experienced professional, not only to assess an environment but also to potentially monitor progress and ameliorate any dangers of the rehabilitation process.

The distributed nature of the domestic environment then raises a challenge for the provision at scale of sufficient domestic visits to support safe and effective use of technologies, and these challenges might be particularly difficult to resolve in areas that are geographically isolated (such as rural communities) [34]. How to address the challenge of providing sufficient in-person support for rehabilitation technology deployment is an open question and one that would need to be addressed by health services as part of the long-term work of integrating interactive technologies into the rehabilitation process.

## Discussion

### Overview

We have described four challenges that affect the design and deployment of effective home-based rehabilitation technologies for stroke. Our aim is to support effective deployment work in the future and therefore the successful uptake of rehabilitation technologies by health services, as a possible route toward recovery from disability acquired through brain injury. The following are three implications arising from these challenges. They were selected to provide insight into how rehabilitation technology deployments can be supported on a larger scale.

### Skilled Workers May Be Needed to Enable Successful Technology Deployments

Although our interest is in rehabilitation technology, much of the complexity we have observed relates to what might be described as human factors around the technology [35], for example, the interactions between people and others in relation to the technology and the interactions between people and the technology itself. For example, the complexity of finding a place for a rehabilitation technology in a home where routines have been disrupted by a brain injury, where traumatic effects of the brain injury persist, and where space is jointly managed by multiple residents likely needs, in many cases, a skilled worker to negotiate; however, some potential users of technology may be able to negotiate these challenges by themselves.

If deployment work is to be done as part of health care systems, and as domestic rehabilitation technologies are a relatively new phenomenon, we might speculate that existing health care professions are, as a whole, unlikely to have the skills or knowledge required to immediately engage in effective deployment work and to negotiate all the challenging human factors identified earlier. In some cases, it seems likely that existing professions may be able to adapt; during deployment studies, we found that a team of physiotherapists were capable of monitoring usage and suggesting alterations to support successful and safe engagement, and we might expect a reasonable match with skills and knowledge present in professions such as occupational therapy.

Regardless of whether rehabilitation technology deployments are supported by health care workers drawn from existing professions or by a new type of health care worker, it seems clear that new tools and potentially new forms of training might be needed to prepare such workers to perform the challenging work of selecting, deploying, and supporting rehabilitation technologies, and provision may be a requirement for the large-scale integration of rehabilitation technologies into real-world health care practice.

### Systematizing the Mapping of the Home May Be Beneficial

One mechanism for supporting health care workers to perform technology deployment work may be evidence-based tools to systematize the mapping of the home and its routines, so as to enable a more rapid understanding of the complexities of this environment than that achieved by our study team. Identifying what features to map in the home of a stroke survivor and collecting mapping information took a significant amount of effort and discussion during our design case studies among a group of researchers with a substantial body of expertise in technology design and deployment. This level of effort would be impractical to repeat on a larger scale, but a strength of the Research through Design approach is that it allows for lessons learned through design-oriented research endeavors to be considered as a primary output of the research process, so as to support the work of others engaging in a similar space.

Our work suggests the importance of considering prior usage of the home (ie, before a stroke occurred); current and anticipated or hoped for future usage of the home; changes in emotional response due to stroke; any spaces purposefully created to support the well-being of a stroke survivor; social usage of a space that might have an impact on the technology integration process; and the availability of utilities necessary to support technologies, principally power sockets but potentially also access to a network. The latter can be affected by structural features such as thick boundary walls or the location of domestic routers. Tolmie et al [24] provided an account of the challenge of deploying network-enabled technologies into domestic networking environments.

If rehabilitation technologies are to be deployed on a wide scale across thousands of homes, then we would argue that mapping exercises, although vital, need to be completed quickly and efficiently. This seems to be an ideal candidate for the creation of standardized materials that systematize the mapping process and allow for the collection of information known to be useful in supporting technology deployment work. This would not exclude human judgment in the final decisions made around rehabilitation technology deployments; however, it would provide for a good foundation of knowledge to be collected to ensure that key decisions around place could be taken efficiently, hopefully increasing the scalability of the process of deploying rehabilitation technologies.

How these standard measures might be made available to people doing technology deployment work, and how deployment specialists might be supported in quickly collecting and analyzing collected information, is a question for further investigation. However, we might imagine the creation of computer interfaces to support the collection of data in the field, as has been done in other areas involving specialist visits to particular locations, such as in the railway maintenance industry [36].

### Education Is a Viable Focus for Rehabilitation Technologies

Much of the technology rehabilitation literature is built around the design and deployment of technologies that encourage rehabilitation by showing people what to do in terms of exercises and by helping them do it, often in a repetitive manner. However, work on Motivating Mobility suggests that teaching people how to understand and conceptualize their rehabilitation is an important role for technology and might boost outcomes. This is very clear in the narrative presented in relation to challenge 3, where 2 partners had such a profound difference of view about how to use a technology. It was also embedded in a broad range of discussions with participants, who often described being motivated to do rehabilitation exercises but not knowing what was appropriate or safe to do and hence not engaging in them.

Providing education about rehabilitation, in this case, from common mental health problems such as anxiety and depression, has been a core approach in the category of health technologies known as Computerized Cognitive Behavioral Therapy; examples of these technologies have been proven to work in large clinical trials [37]. We might speculate about the possible efficacy of deploying technologies that provide education in the principles of rehabilitation.

### Conclusions

The design of interactive technologies to support rehabilitation from disability acquired through brain injury has been a topic of research since 1991, and hundreds of technology prototypes have been piloted and reported in the literature, with the domestic environment being a focus of research. We have conducted research work intended to support an understanding of how domestic rehabilitation technologies might be integrated into health care practice and considered a range of human factors at play in this process.

We suggest that for large-scale deployments to become a practical reality, the preparation of health care professionals needs to be considered, and health care professionals need to be provided with appropriate tools (such as systematized, evidence-based methods to allow for the mapping of deployment environments).

Deployments may also need to consider the provision of education for the recipients of technology (including both brain injury survivors and others in their social context), as a lack of knowledge of rehabilitation and how it can occur may be a barrier to engagement with the deployed technology.
