# Correction: Homes of Stroke Survivors Are a Challenging Environment for Rehabilitation Technologies

**DOI:** 10.2196/32418

**Published:** 2021-08-16

**Authors:** Stefan Rennick-Egglestone, Sue Mawson

**Affiliations:** 1 School of Health Sciences Institute of Mental Health University of Nottingham Nottingham United Kingdom; 2 School of Health and Related Research University of Sheffield Sheffield United Kingdom

In “Homes of Stroke Survivors Are a Challenging Environment for Rehabilitation Technologies” (JMIR Rehabil Assist Technol 2021;8(2):e12029) the authors noted three errors.

The Acknowledgments section of the paper has been updated to effectively acknowledge the work of the broader Motivating Mobility team. In the originally published version, the Acknowledgements section read as follows:

This work was supported by the Engineering and Physical Sciences Research Council (grants EP/F00382X/1, EP/F03038X/1, and EP/M000877/1).

This has been updated to:

This work was supported by the Engineering and Physical Sciences Research Council (grants EP/F00382X/1, EP/F03038X/1, and EP/M000877/1). Our article reflects on the body of work produced by the Motivating Mobility team from 2007 to 2010. The authors would like to acknowledge the contribution of all team members to this body of work. Motivating Mobility was a collaboration between the University of Sussex (Lesley Axelrod, Madeline Balaam, Eric Harris, Geraldine Fitzpatrick), the University of Southampton (Ruth Turk, Ann-Marie Hughes, Jane Burridge), Sheffield Hallam University (Anna Wilkinson, Sue Mawson), the University of Oxford (Nour Shublaq, Penny Probert Smith), the University of Nottingham (Stefan Rennick-Egglestone, Tom Rodden), and the University of Dundee (Thomas Nind, Ian Ricketts).

The captions of 3 figures have been updated to clarify that the images were drawn from previous articles cited in the paper. The following text has been added to the original caption of Figures 1 and 2:

First published in the study by Axelrod et al [26].

The following text has been added to the original caption of Figure 4:

First published in the study by Balaam et al [30].

In the originally published paper, the following sentence was included in the “Challenge 1: Identifying a Location for a Rehabilitation Technology” section:

Early in Motivating Mobility, we conducted a photographic study of the homes of stroke survivors recruited by the project.

The text may have inadvertently created the impression that one author (SR-E) was involved in a Motivating Mobility study for which he was in fact not involved. For clarity, this sentence has been updated to:

Early in Motivating Mobility, a photographic study of the homes of stroke survivors was conducted [26].

The correction will appear in the online version of the paper on the JMIR Publications website on August 16, 2021, together with the publication of this correction notice. Because this was made after submission to PubMed, PubMed Central, and other full-text repositories, the corrected article has also been resubmitted to those repositories.

